# Job specific health status of workers in ayurvedic pharmaceutical manufacturing units across Kerala: a cross-sectional study

**DOI:** 10.3389/fpubh.2026.1781213

**Published:** 2026-03-11

**Authors:** Pournami V. U, Meera S., Tony Jacob John, Vandana Rani M., Anusree Dileep, Delvin T. Robin

**Affiliations:** Department of Swasthavritta (Social and Preventive Medicine), Amrita School of Ayurveda, Amritapuri, Amrita Vishwa Vidyapeetham, Kollam, India

**Keywords:** Ayurvedic pharmaceutical industry, environmental exposure, ergonomic risk factors, manufacturing worker, occupational hazards, occupational health

## Abstract

**Introduction:**

Workers in small and medium scale Ayurvedic pharmaceutical manufacturing units are exposed to various occupational hazards like heat, fumes, dust, noise, and strenuous physical activities due to reliance on traditional production methods. Evidence on job specific health risks in this sector remains limited. The objective of this study was to know the current health status of workers in Ayurvedic manufacturing units.

**Methods:**

A cross-sectional study was conducted among 152 workers employed in small and medium-scale Ayurvedic pharmaceutical industries across Kerala using a structured questionnaire of 25 items to assess their physical(13 items), mental(5 items) and environmental(7 items) health status. The data were collected by the principal investigator via face-to-face interview. Sociodemographic and occupational characteristics were also recorded. Association between health outcomes and job roles was analyzed using chi-square test.

**Results:**

Physical and environmental health outcomes showed strong association with job roles. Workers involved in production, packing, milling, grinding, and raw material store reported higher prevalence of tiredness, nasal complaints, skin allergy, body pain, prolonged standing or sitting, repetitive hand movements, heavy lifting and exposure to environmental conditions like dust, heat, noise, vibration, sunlight, and smoke. In contrast, mental health outcomes like perceived stress, difficulty in maintaining concentration, mood changes and sleep disturbances did not demonstrate significant association with job roles and were more evenly distributed among the workforces.

**Conclusion:**

These findings demonstrate that task related ergonomic demand and environmental exposure are the primary drivers of occupational morbidity in an Ayurvedic pharmaceutical manufacturing unit. These findings also highlight the need for task specific ergonomic and environmental intervention to improve worker health and promote safer and more sustainable practices in Ayurvedic pharmaceutical industries.

## 1 Introduction

Occupational health is a core component of public health and is primarily concerned with promotion of physical, mental and social wellbeing of workers in all occupations and emphasizes on the prevention of workplace hazards at a primary level (1).

Healthy workers contribute to the wellbeing of their families and positively influence productivity and efficiency within the workplace and society (2). An occupational hazard refers to any workplace experience such as unplanned and damaging event that disrupts an activity and is always the result of unsafe actions, unsafe conditions, or a combination of both (3).

Workers in all professions are exposed to hazardous elements which place their health at risk, such as carrying heavy loads, environmental and psychological stresses, exposure to dangerous chemicals, heat, unsafe machinery, noise, and dust (4, 5). These exposures may result in occupational illnesses or exacerbate pre-existing health conditions (6).

In India, major occupational diseases include pneumoconiosis (like silicosis, bagassosis, anthracosis, and byssinosis), musculoskeletal disorders, noise-induced hearing loss, pesticide poisoning, and occupational accidents, particularly among workers in manufacturing and industrial sectors (7).

The pharmaceutical sector serves as the backbone of the healthcare system. Since healthcare items must be manufactured under strict circumstances, this industry is typically characterized by an environment with quality levels that are normally higher than the other manufacturing sectors (8). Despite these controls, workers in pharmaceutical industries are exposed to high heat, fumes, dust, humidity, and noise and suffer adverse health outcomes such as occupational asthma, pharmacological effects, reproductive problems, and dermatitis due to occupational exposures (9).

Ayurvedic pharmaceutical industry represents a unique and comparatively understudied manufacturing sector. Ayurvedic pharmaceutical manufacturing differs from conventional pharmaceuticals in several process-level characteristics. Conventional manufacturing pre-dominantly relies on standardized chemical inputs, closed processing systems, and automated unit operations designed to minimize direct worker exposure (10). In contrast, small and medium scale Ayurvedic manufacturing units are labor intensive (11). These units include processes involving handling and processing medicinal substances of plant, mineral, metal, and animal origin, carried out through mechanical or manual methods. Workers in small and medium-scale Ayurvedic pharmaceutical units are frequently exposed to high temperatures, fumes, dust, humidity, noise, and strenuous physical activities such as lifting heavy loads, continuous stirring, pill rolling, prolonged standing, and repetitive hand movements. These processes distinctly create an occupational condition unique to Ayurvedic pharmaceutical workers. Since workers' health is crucial to productivity and quality of healthcare products, this study was undertaken as a cross-sectional survey among workers in small and medium-scale Ayurvedic pharmaceutical industries across Kerala to assess their physical health status, mental health status, and workplace environmental conditions.

The World Health Organization highlights the widespread use and policy relevance of traditional medicine worldwide and has prioritized integration and quality assurance of traditional systems, including Ayurveda (12, 13). Policy analysis estimate that Indian AYUSH industry has reached approximately USD 18.1 billion in 2020 and has demonstrated sustained growth in recent years, underscoring the increased scale of production and workforce involvement in this sector (14).

In summary, the objectives of this study were to assess the current occupational health status of workers employed in small and medium-scale Ayurvedic pharmaceutical industries and to assess the physical health, mental health, and the workplace environmental conditions of workers in manufacturing unit.

## 2 Methods

### 2.1 Study design and study setting

A cross-sectional survey was conducted among workers of small and medium scale Ayurvedic pharmaceutical industries across Kerala. Workers from different sections of the manufacturing process, including production, packing, and raw material handling units, were included in the survey. This study was approved by the Institutional Ethics Committee of Amrita School of Ayurveda, Kollam, Kerala, India (Ethics No: IEC.ASA.PGR.06, dated 02/11/2023). All procedures were carried out in accordance with the relevant ethical guidelines and regulations.

### 2.2 Study population and eligibility criteria

This study comprised of workers employed in selected small and medium scale Ayurvedic pharmaceutical manufacturing units across Kerala. According to the 13th Five-Year Plan (2017–2022) working Group on AYUSH report (Govt. of Kerala-Kerala State Planning Board), approximately 850 Ayurvedic pharmaceutical units operate in Kerala, of which around 20 are considered small- and medium-scale sector. Each of these eligible units was considered as a cluster. From these clusters, five Ayurvedic pharmaceutical manufacturing units were selected using a lottery method, and workers within the selected units were recruited using convenience sampling. Of the total 152 participants, 30 were recruited from Unit one, 15 each from Unit two, three and four, and 77 from Unit five, based on workforce size and availability of workers during collection. Workers between the ages of 20–60 years who have been employed in the concerned department for more than 3 years and involved in manufacturing process area and raw material store area were included in the study. Information on pre-existing medical conditions were obtained during recruitment, and workers who self-reported medical conditions that could affect the measured outcomes were excluded. Workers who were unwilling to participate were also excluded.

### 2.3 Sample size and sampling technique

The sample size was estimated based on the prevalence obtained from a pilot study conducted by the investigators among workers in small scale Ayurvedic pharmaceutical manufacturing unit. Using a prevalence of 41% and an absolute precision of 5%, the minimum required sample size was calculated. A total of 152 eligible workers were included in the study.

### 2.4 Study tool/questionnaire

The questionnaire used in this study was newly developed by the research team specifically for the present study and was not adopted from any previously published instrument. The data was collected using a structured, closed ended questionnaire developed and validated for the present study. It comprised of 25 items organized under three domains: physical health status (13 items), Mental health status (five items), Environmental status (seven items). Responses were recorded using a five-point Likert scale. The tool also included items on sociodemographic and occupational details. The full questionnaire is provided in Supplementary material (Data Sheet 1). Questionnaire development initially consisted of 26 items, which were developed based on literature review and inputs from experts. Item development was guided by occupation health literature relevant to industrial and pharmaceutical work setting, with emphasis on physical, mental, and environmental work-related conditions. The items were translated into local language prior to validation. Face validity was assessed by eight subject experts, who evaluated each item for clarity, understanding, unambiguity, and likelihood of misinterpretation. Based on face validity assessment, one item was deleted, four items were modified, and one item was formatted into three questions.

Subsequently, content validity was assessed by five content experts with expertise in Ayurveda. Scale level and Item level content validation index were done, following which additional two items were removed. A pilot study using the revised 25-item questionnaire was then conducted among 30 workers from a small-scale Ayurvedic pharmaceutical unit to identify ambiguities and finalize the tool.

Internal consistence reliability of the final questionnaire was assessed using the Cronbach's alpha coefficient. All three domains demonstrated acceptable internal consistency, with Cronbach's alpha values greater than 0.7, indicating good reliability of the questionnaire. Validation included face and content validity, followed by pilot testing. Construct validity was assessed using Exploratory Factor Analysis and confirmed through Confirmatory Factor Analysis. Detailed results of the exploratory and confirmatory factor analyses are presented in Supplementary material (Data Sheet 2). Internal consistency reliability was evaluated, and Structural Equation Modeling was applied to examine latent construct relationships and overall model fit for descriptive assessment.

### 2.5 Survey administration and data collection procedure

Data was collected through face-to-face interview at selected Ayurvedic pharmaceutical manufacturing units across Kerala. The survey was administrated by the investigator using the validated questionnaire. Prior to data collection, purpose of the study was explained and written informed consent was collected.

Participants were interviewed during their working hours without interfering their routine work activities. No personal identifiers were recorded to ensure confidentiality. The completed questionnaires were checked for completeness on the same day of data collection. Data was collected over a period of 18 months, from April 2023 to September 2024.

### 2.6 Statistical analysis

Data were entered, cleaned, and analyzed by SPSS (version 26.0). Descriptive statistics were used to summarize sociodemographic characteristics, occupation profiles, and response related to physical health, mental health, and workplace environmental conditions. Results were expressed as frequency, percentage, charts and graphs. Associations were assessed using chi-square test, *p*-value of less than 0.05 was considered statistically significant.

## 3 Results

### 3.1 Sociodemographic characteristics of workers

A total of 152 workers from medium and small scale Ayurvedic pharmaceutical manufacturing unit were included in the survey. The largest age group were within the ages of 41–50 years (32.24%) and 31–40 years (30.26%), followed by 51–60 years (23.68%) and 21–30 years (13.82%). The sex distribution was balanced, with 50.66% females and 49.34% males. Most participants were married (84.21%), while 13.82% were unmarried and 1.97% were widowed. The majority identified as themselves as Hindu (71.05%), followed by Christian (21.71%) and Muslim (7.24%). Based on BMI, 56.58% of workers were overweight, 5.26% were obese, 2.63% were underweight and 35.53% were within normal BMI. Employment included those in production (29.61%), packing (28.29%), store (11.18%), raw-material store (9.21%), tablet section (9.21%), mill (8.55%), grinding (3.29%) and boiler work (0.66%). Work experience ranged from 0 to 49 years, with 30.26% of subjects with 0–7 years of experience, 27.63% with 8–14 years, 22.37% reporting 15–21 years, 8.55% with 22–28 years, 5.26% with 29–35 years, 3.95% with 36–42 years and 1.97% stated 43–49 years of job experience. At pre-placement medical assessment, 3.29% of workers reported a history of allergy and 96.71% reported no prior medical complaints. The full distribution is presented in Table 1.

**Table 1:** Socio-demographic and occupational characteristics of the study participants.

**Variable**	**Category**	**Frequency**	**Percentage**
Age group (in years)	21–30	21	13.82
	31–40	46	30.26
	41–50	49	32.24
	51–60	36	23.68
Sex	Female	77	50.66
	Male	75	49.34
BMI (Kg/m^2^)	Underweight (< 18.5)	4	2.63
	Normal weight (18.5–24.9)	54	35.53
	Overweight (25–29.9)	86	56.58
	Obese (>30)	8	5.26
Job role	Boiler	1	0.66
	Grinding	5	3.29
	Mill	13	8.55
	Packing	43	28.29
	Production	45	29.61
	Raw material store	14	9.21
	Store	17	11.18
	Tablet section	14	9.21
Job experience (in years)	0–7	46	30.26
	8–14	42	27.63
	15–21	34	22.37
	22–28	13	8.55
	29–35	8	5.26
	36–42	6	3.95
	43–49	3	1.97
Preplacement data	Allergy	5	3.29
	Nil	147	96.71

### 3.2 Associations between sociodemographic variables and questionnaire items

Chi-square analysis helped identify several statistically significant associations with Job role, such as tiredness, nasal complaints, allergies/skin disease, suppression of urges, pain during work, prolonged standing, repetitive hand movements, lifting heavy loads, prolonged sitting, noise exposure, raising voice, mechanical vibration, heat exposure, sun exposure, dust exposure, and smoke exposure all having *p* < 0.05. The complete *p*-value distribution is shown in Table 2.

**Table 2:** Associations between health related questionnaire items and job roles.

**Domain**	**Sl. No**.	**Items**	**Degree of Freedom (df)**	**Job**	
				**Chi-Square**	***p*** **-value**
Physical	1.	Tiredness	7	21.50	0.00
	2.	Nasal complaints	7	23.68	0.00
	3.	Respiratory problems	7	12.50	0.1
	4.	Allergies or skin diseases	7	24.49	0.00
	5.	Eye irritation	7	8.05	0.32
	6.	Suppression of natural urges	7	15.41	0.03
	7.	Body pain	7	22.99	0.00
	8.	Prolonged standing	7	40.99	0.00
	9.	Repetitive hand movement	7	75.50	0.00
	10.	Weight lifting	7	42.31	0.00
	11.	Prolonged sitting	7	79.07	0.00
	12.	Bending	7	7.76	0.4
	13.	Absenteeism	7	3.49	0.8
Mental	14.	Perceived stress	7	8.84	0.3
	15.	Difficulty in maintaining focus	7	5.19	0.63
	16.	Moodiness	7	11.89	0.10
	17.	Disturbed sleep	7	4.17	0.75
	18.	Difficulty in falling asleep	7	8.31	0.30
Environmental	19.	Perceived exposure to noise	7	22.41	0.00
	20.	Perceived raise voice at work	7	29.47	0.00
	21.	Perceived exposure to mechanical vibrations	7	33.80	0.00
	22.	Perceived exposure to temperature	7	87.87	0.00
	23.	Perceived exposure to sun light	7	28.07	0.00
	24.	Perceived exposure to dust	7	18.25	0.01
	25.	Perceived exposure to smoke	7	94.38	0.00

Chi-square test applied; *p* < 0.05 considered statistically significant.

### 3.3 Job wise and physical health status

Workers reported multiple physical complaints, including tiredness, nasal complaints, respiratory discomfort, allergy or skin problems, irritation of eyes, suppression of urges, pain, prolonged standing or sitting, repetitive movements, carrying heavy load or bending.

Workers in production reported higher tiredness score with a median (IQR) of 3 (2–3), compared with boiler and store workers, who demonstrated lower medians of 2 (1–2). Nasal complaints were common among workers in mill and grinding with median score of 3 compared to store, production, and raw-material store units with median responses of 2 (1–3) across these sections. Grinding workers showed the highest burden of allergies or skin diseases, with a median of 4 (3–4), compared to other job roles which reported lower median scores of 3 (1–3). Suppression of urinary or bowel urges were frequently reported in tablet section and boiler workers with median scores of 4 (3–4) compared to packing, store, production, raw-material store, and grinding workers with median of 3.

Pain during work demonstrated a clear variation among all job groups. Tablet-section workers reported the highest median pain scores with median 4, while packing and production workers reported lower frequency of pain with median 3, with some individuals reporting persistent pain at the upper end of the scale. Ergonomic-task requirement such as prolonged sitting/standing, manual lifting was work dependent. Frequent prolonged standing was reported among production and mill workers, with median values of 4 (3–4). Repetitive hand movements were frequently reported by workers involved in packing, store, production, mill, tablet section, raw material store and grinding, all demonstrating higher median scores of 4 (3–5). Manual lifting of heavy loads was reported more frequently by mill and raw material store workers, with median 4 (3–4), compared with store, production, grinding and boiler workers who reported lower median of 3 (2–4). Prolonged sitting related task was frequently reported among tablet-section workers, with median score of 4 (4–5), followed by packing with median 3 (3–4), whereas mill and grinding workers reported minimal sitting-related demands.

Physical health items like respiratory problems, eye irritation, bending at work and absenteeism did not show a statistically significant association with job role. Job-wise median and interquartile range (IQR) values for all questionnaire items are provided in Table 3.

**Table 3:** Job-wise median (IQR) scores of physical, mental, and environmental health domain items.

**Domain**	**Sl. No**	**Items**	**Job**							

			**Packing**	**Store**	**Production**	**Mill**	**Tablet Section**	**RM Store**	**Grinding**	**Boiler**
Physical	1	Tiredness	1 (1–2)	2 (1–2)	3 (2–3)	1 (1–1)	1 (1–2)	1 (1–1)	1 (1–2)	2 (2–2)
	2	Nasal complaints	1 (1–2)	2 (1–3)	2 (1–3)	3 (3–3)	1 (1–2)	2 (1–3)	3 (2–3)	1 (1–1)
	3	Respiratory problems	1 (1–1)	1 (1–3)	1 (1–2)	2 (2–3)	1 (1–1)	1 (1–3)	2 (1–2)	2 (2–2)
	4	Allergies or skin diseases	1 (1–2)	1 (1–2)	1 (1–2)	3 (1–3)	3 (1–3)	3 (2–3)	4 (3–4)	1 (1–1)
	5	Eye irritation	1 (1–2)	1 (1–3)	2 (1–3)	2 (2–3)	1 (1–1)	1 (1–2)	2 (1–3)	3 (3–3)
	6	Suppression of natural urges	3 (2–4)	3 (2–3)	3 (1–3)	2 (1–2)	4 (3–4)	3 (2–4)	3 (3–3)	4 (4–4)
	7	Body pain	3 (2–3)	2 (1–3)	3 (2–3)	1 (1–2)	4 (4–4)	2 (1–3)	2 (1–2)	1 (1–1)
	8	Prolonged standing	3 (1–3)	3 (3–3)	4 (3–4)	4 (3–4)	2 (1–3)	3 (3–3)	3 (3–3)	3 (3–3)
	9	Repetitive hand movement	4 (4–5)	4 (3–4)	4 (4–5)	4 (3–4)	4 (4–5)	4 (3–4)	4 (4–4)	3 (3–3)
	10	Weight lifting	2 (1–3)	3 (2–4)	3 (3–4)	4 (3–4)	2 (2–2)	4 (3–4)	3 (3–3)	3 (3–3)
	11	Prolonged sitting	3 (3–4)	2 (1–3)	1 (1–2)	1 (1–1)	4 (4–5)	2 (1–3)	1 (1–1)	2 (2–2)
	12	Bending	4 (3–4)	4 (3–4)	4 (3–4)	4 (3–4)	4 (3–4)	4 (3–4)	4 (3–4)	3 (3–3)
	13	Absenteeism	2 (1–3)	2 (1–2)	2 (1–3)	2 (1–3)	3 (2–3)	2 (1–2)	2 (1–3)	2 (2–2)
Mental	14	Perceived stress	1 (1–2)	1 (1–2)	2 (1–3)	2 (1–3)	2 (1–2)	1 (1–1)	1 (1–1)	3 (3–3)
	15	Difficulty in maintaining focus	1 (1–2)	1 (1–2)	1 (1–2)	1 (1–1)	2 (1–2)	1 (1–1)	1 (1–3)	1 (1–1)
	16	Moodiness	2 (1–3)	2 (2–3)	3 (2–4)	2 (2–3)	3 (2–3)	2 (1–3)	2 (1–3)	3 (3–3)
	17	Disturbed sleep	1 (1–2)	2 (1–3)	2 (1–3)	2 (1–2)	2 (1–3)	1 (1–2)	1 (1–4)	3 (3–3)
	18	Difficulty in falling asleep	1 (1–2)	1 (1–2)	2 (1–2)	2 (1–2)	1 (1–2)	1 (1–2)	3 (1–3)	4 (4–4)
Environmental	19	Exposure to noise	3 (2–3)	2 (1–2)	3 (2–3)	4 (4–5)	3 (1–3)	2 (2–3)	5 (5–5)	3 (3–3)
	20	Raise voice at work	2 (1–3)	1 (1–2)	2 (1–3)	4 (4–4)	2 (1–3)	1 (1–2)	4 (4–4)	3 (3–3)
	21	Exposure to mechanical vibrations	3 (2–3)	1 (1–2)	2 (1–3)	5 (4–5)	3 (3–4)	1 (1–2)	5 (4–5)	4 (4–4)
	22	Exposure to temperature	1 (1–2)	2 (1–2)	4 (3–4)	2 (1–2)	2 (1–2)	2 (1–2)	1 (1–3)	4 (4–4)
	23	Exposure to sun light	1 (1–2)	3 (2–3)	2 (2–3)	2 (2–3)	3 (2–3)	3 (2–3)	3 (3–3)	3 (3–3)
	24	Exposure to dust	3 (2–4)	3 (3–4)	3 (2–3)	5 (4–5)	3 (3–3)	4 (3–4)	4 (4–5)	2 (2–2)
	25	Exposure to smoke	1 (1–1)	1 (1–1)	3 (3–4)	1 (1–1)	1 (1–2)	1 (1–2)	1 (1–2)	3 (3–3)

### 3.4 Job wise and mental health status

Five items were assessed under mental-health domain, including excessive stress during work, difficulty in maintaining concentration, mood or temper outbursts, disturbed sleep, and difficulty in falling asleep. Across all job categories, median (IQR) values remained relatively uniform, indicating minimal variation in mental-health responses by work role.

None of the mental-health domain items showed statistically significant association with job in the chi-square analysis (*p* > 0.05). Workers across production, packing, storage, milling, grinding, tablet, and boiler sections reported comparable levels of stress, sleep disturbance, concentration difficulty, and mood-related symptoms.

### 3.5 Job wise and working environment status

Seven items assessed the environmental and sensory exposure, including need to raise their voice at work, and perceived exposure to noise, mechanical vibrations, temperature, sunlight, dust and smoke. Environmental and sensory exposure differed across different work areas with clear variations in median (IQR) scores.

Frequent exposure to high noise was self-reported among workers of grinding, with median of 5 followed by mill workers with median of 4. Raising one's voice was commonly self-reported among workers of mill and grinding, with median values of 4 (4–4). Workers in mill and grinding section perceived to be most affected by mechanical vibrations, demonstrating higher median of 5 (4–5) compared with other job roles. Heat exposure was perceived to be higher among workers of production and boiler, median 4 (3–4). Store, tablet section, raw material store, grinding and boiler workers self-reported that they were exposed to sunlight frequently, with median scores of 3 (2–3). Higher dust exposure was perceived by workers of mill, with median score of 5 (4–5) followed by raw material store and grinding section, median score of 4 (3–5), while smoke exposure was pre-dominantly self-reported among production and boiler workers, with a median of 3 (3–4). Job-wise median and interquartile range (IQR) values for all questionnaire items are provided in Table 3.

## 4 Discussion

This cross-sectional survey assessed the job-specific patterns of self-reported occupational health complaints among workers in small and medium scale Ayurvedic pharmaceutical manufacturing units using a structured questionnaire covering physical health, mental health, and environmental domains. The findings indicate that self-reported physical and perceived environmental health complaints varied across job roles, whereas mental health related symptoms did not show a clear association with job category, suggesting a domain specific variation in the reported outcomes.

### 4.1 Physical health status

Physical health domain showed the most association with job roles. Worker reported multiple self-reported physical complaints, including tiredness, nasal complaints, allergy or skin irritation, suppression of bladder and bowel urges, pain, repetitive hand movements, frequent bending, prolonged standing and sitting. These findings suggest that the distribution of reported physical health complaints in a workplace such as Ayurvedic pharmaceutical units is job specific (15).

#### 4.1.1 Tiredness

Production workers reported the highest self-reported tiredness scores among all the job categories. This pattern may reflect the physical workload involved in manufacturing Ayurvedic medicines such as drying, mixing, boiling, frying, and manual handling of medicines. These activities require prolonged standing (16), lifting weight, often with exposure to heat, dust and smoke. Previous occupational health studies described that prolonged workload and static postures as contributors to perceived occupational fatigue and reduced workplace capacity (17).

#### 4.1.2 Nasal complaints

Perceived nasal complaints were most frequently reported by workers of mill and grinding sections. These areas are generally involved in grinding and handling powdered herbs, raw drugs, and finished products, results in generation and increased exposure to airborne dust and particulate matter. Herbal dust may act as potential irritants or allergens, and repeated inhalation may cause irritation of the upper respiratory tract and nasal mucosa, leading to symptoms such as congestion, irritation, and discomfort (18, 19). The concentration of nasal complaints in these job roles highlights the importance of particulate control and respiratory protection in Ayurvedic pharmaceutical settings.

#### 4.1.3 Allergy or skin disease

The highest frequency of allergy or skin irritation was self-reported among grinding workers. This may be due to repeated contact with raw herbal materials and fine powders during grinding process. Medicinal plants used in Ayurveda may contain phytochemicals such as alkaloids, phenolics, resins, or essential oils that are known occupational sensitizers (20, 21). Prolonged dermal exposure, especially in the absence of protective gloves, has been associated with skin related symptoms, such as contact dermatitis and allergic skin reactions and reinforces the role of material handling as a key determinant of dermatological morbidity.

#### 4.1.4 Suppression of urges

A significant portion of workers reported they often suppressed urinary and bowel urges, particularly those in tablet and boiler section. This suggests that workplace factors may be influencing bladder and bowel health. Prolonged suppression can contribute to urinary tract infections, constipation, or other digestive issues (22). A rigid workplace may make it more difficult for employees to take care of their physical demands/bodily needs. This problem could be exacerbated by jobs that require physically demanding tasks, long hours, or restricted access to bathrooms. Chronic suppression may lead to discomfort, pain, and potentially more serious health problems. This behavior can also be related to professional activities of working women. For example, wearing particular clothing, working in hot or cold temperatures, and stressful job demands can limit the ability to go to the bathroom when needed (23). However, in the present study findings represents self-reported behavior and does not indicate clinically diagnosed urogenital or gastrointestinal conditions. So, educating workers about the problems associated with suppression of urges and providing regular breaks can help protect workers' health in the surveyed units.

#### 4.1.5 Pain while work

Self-reported pain during work was frequently reported among several job roles, with tablet-section workers demonstrating the highest median pain scores. Some workers from packing and production workers also reported persistent pain at higher frequencies, with some indicating that some workers always experienced pain. This may be due to the ergonomic demands of these roles which requires the workers to stay in unhealthy postures, repetitive hand movements, prolonged standing, bending, and manual handling of materials. Sustained exposure to such risk factors may increase mechanical stress on muscles, joints, and connective tissues, which may cause cumulative trauma and chronic pain, leading to work-related musculoskeletal disorders (WMSDs) (24, 25).

#### 4.1.6 Prolonged standing and sitting

Prolonged standing was most frequently reported in production, and mill areas, while prolonged sitting was more common in tablet sections and packing. Extended static postures, whether standing or sitting, impair blood circulation and increase muscular load, particularly in the lower limbs, back, and neck. Over time, these postural stresses contribute to discomfort, perceived fatigue, and musculoskeletal disorders (26–28). The coexistence of prolonged standing and sitting across different job roles highlights the need for task rotation and posture variability within the workday.

#### 4.1.7 Repetitive hand movements

Tasks requiring repetitive hand and wrist movements were common in Ayurvedic pharmaceutical industry and frequently reported among workers of packing, store, production, mill, tablet section, raw material store and grinding. In the Ayurvedic pharmaceutical industry, traditional practices such as manual pill rolling require continuous fine motor activity involving fingers and wrists. Occupational health literature has described any muscle group will ultimately become fatigued from repetitive motions, which may contribute to tension in other muscles as the activity becomes more difficult, pre-disposing workers to conditions such as tendinitis and carpal tunnel syndrome (29). Although tablet compression machine is used in some pharmaceutical industries for tablet-making processes, [which is dust free also (30)], some still rely on manual methods, i.e. hand rolled tablets thereby sustaining ergonomic risk.

#### 4.1.8 Weight-lifting

Manual handling and lifting of heavy loads were mostly reported among mill and raw material store workers. Tasks involving lifting, pushing, pulling, and frequent bending are associated with biomechanical stress on the spine and lower extremities. Improper lifting techniques and excessive load weights increase the risk of acute injuries and chronic musculoskeletal disorders, mainly lower back pain (31, 32). Ergonomic intervention such as manual lifts of loads weighing more than 10 kg should be avoided, introducing lifting aids for all lifts weighing more than 10 kg. Other measures are reducing the weights of boxes, use of height-adjustable rack; avoid use of unfavorable storage heights; reduce push–pull distance; reduce the loading–unloading pace; provide additional worker for sharing the load. Thus, it can be helpful to reduce the risks associated with these activities by suggesting interventions that emphasize safe lifting practices, mechanization of tasks, and the provision of personal protection equipment (33, 34).

### 4.2 Mental health status

The mental health domain assessed perceived stress, difficulty maintaining concentration, mood disturbances, disturbed sleep, and difficulty falling asleep. None of the mental health items demonstrated a statistically significant association with job role in the present study. Although symptoms such as stress and sleep disturbance were reported among workers, their distribution appeared relatively uniform across job roles, rather than being driven by job specific occupational demands. This pattern is consistent with occupational health literature, which describes that mental health outcomes are influenced by psychosocial work environment, organizational factors, individual coping capacity, and non-occupational stressors rather than by physical job demands alone (35). Sleep disturbances among working populations has similarly been linked to age-related changes, cumulative stress, and underlying health conditions rather than to specific manual tasks (36). The absence of job-specific clustering in mental health outcomes in the present study does not imply the absence of psychological burden, but that mental-health symptoms in this workforce may be shaped by personal, social, and non-occupational factors and may also be underreported in occupational surveys due to stigma or normalization of stress.

### 4.3 Environmental status

Environmental status demonstrated strong associations with job role. This suggests that exposure to perceived environmental stressors, such as, noise, mechanical vibration, heat, sunlight, dust, and smoke in Ayurvedic pharmaceutical manufacturing units is highly task specific.

#### 4.3.1 Noise and raised voice

In the present study, workers in the grinding an mill sections more frequently reported perceived exposure to high noise levels. While the need to raise one's voice to communicate was commonly reported among those working in mill, and grinding, which may reflect high ambient sound levels. Occupational health literatures has described prolonged exposure to high noise levels has the potential to harm the ear (37). This high ambient noise level may be due to machinery and equipment used in the manufacture of medicines, fans and ventilation systems, air compressors, pumps and water treatment systems, as well as grinding of raw drugs. Studies show that such exposure have a negative impact on the body. Of these, hearing loss is the most prevalent, followed by tinnitus, communication problems, psychological disturbances, physiological effects, and sleep disturbances (38). In order to comply with ergonomic noise standards, workers must wear protective headphones or earplugs because of the high decibel level (39), develop noise monitoring programs and operator exposure assessments, educate employees about the dangers of noise exposure, and foster a safety culture (40).

#### 4.3.2 Mechanical vibration

Self-reported exposure to mechanical vibration was more frequently reported among workers in the mill and grinding sections, likely reflecting the use of machinery in these areas. According to Charles et al. (41) occupational exposures to whole-body or hand–arm vibration was significantly associated with or resulted in musculoskeletal disorders especially in the shoulder and neck. Tasks involving the use of hand-held tools and machinery can expose workers to hand-arm vibration syndrome (HAVS), characterized by numbness, tingling, and pain in the hands and fingers. The quality of life and hand function may be seriously affected by such a condition (42). The health of employees may be safeguarded by taking steps like introducing ergonomic interventions, using materials that absorb vibration, and providing regular breaks.

#### 4.3.3 Temperature

Self-reported exposure to high temperature was more frequently reported among workers in the production and boiler sections, where processes such as boiling, frying, drying, and mixing are commonly performed. In tropical climates, high ambient temperature and humidity in Kerala (43), combined with inadequate ventilation, can exacerbate heat stress and impair work performance (44) and, heat related ailments such as heat cramps, heat stroke, heat syncope, heat rash, heat exhaustion, dehydration, heat fatigue, and hyperthermia can result from the above. Clothing, environmental factors and physical effort all contribute to heat stress. It's equally important to increase employee awareness about the importance of using sunscreen and personal protection equipment while working. In addition, it would be essential to encourage them to report any heat-related complaints and to drink water or other fluids on an intermittent basis.

#### 4.3.4 Sunlight

Perceived sunlight exposure was mainly reported by store, tablet section, raw material store, grinding and boiler workers, reflecting outdoor tasks such as cleaning, washing and drying of raw materials. Ioannou et al. study reported that exposure to the sun may cause heat strain symptoms such weakness, dizziness and impairs cognitive function in both hot and moderate climates. Workers can be protected from adverse effects by encouraging them to wear protective clothes, use sunscreen, and look for shade during the hottest parts of the day (45).

#### 4.3.5 Dust

Significant portion of workers in mill, raw material store and grinding sections reported perceived exposure to dust. This was where milling, grinding, and storage are done which generated airborne particulates. Occupational exposure to airborne contaminants poses inherent health risks, including respiratory disorders and other long-term health implications (46, 47), such as, chronic cough, breathlessness and wheezing among workers who are exposed to dust (48). To address dust-related hazards, both personal protection equipment and dust control must be installed at the milling locations.

#### 4.3.6 Smoke

Smoke exposure was pre-dominantly reported by production and boiler workers, likely due to the continued use of traditional methods of medicine preparation involving wood-burning stoves. Wood smoke exposure has been recognized as a risk factor for lung conditions such chronic obstructive pulmonary disease (COPD) and acute respiratory infections (ARI) (49). This highlights the importance of strict safety measures at workplace, such as offering personal protection equipment and sufficient ventilation.

## 5 Conclusion

This cross-sectional survey describes job-specific patterns of self-reported occupational health complaints among workers in the studied Ayurvedic pharmaceutical manufacturing units. Physical and perceived environmental health outcomes were strongly job-dependent, with distinct clustering of symptoms and exposures according to specific work roles and task demands.

High prevalence of prolonged standing and sitting, repetitive hand movements, pain, and heavy lifting underscores the cumulative ergonomic burden faced by workers in Ayurvedic pharmaceutical units. This is consistent with previous occupational health research, that awkward postures, static positions, and repetitive tasks are associated with musculoskeletal disorders, pain, fatigue, and other physical complaints. This study demonstrates that the nature of work performed is directly linked to physical morbidities as workers engaged in production, packing, milling, grinding, and raw-material handling experienced higher levels of tiredness, nasal irritation, skin allergies, and musculoskeletal strain, reflecting the direct influence of task-specific ergonomic and exposure related stressors.

Perceived exposure to noise, vibration, heat, sunlight, dust, and smoke varied clearly across job roles indicating the need of environmental control measures in traditional manufacturing settings.

In contrast, mental health outcomes did not show a significant association with job roles, suggesting that psychological symptoms were more diffusely distributed across the workforce and may be influenced by individual or non-occupational factors rather than task-specific exposures.

From a public health perspective, these findings indicate that physical workload and perceived environmental exposure are the main causes of health problems among workers of Ayurvedic pharmaceutical manufacturing unit. Although the results are based on self-reported experiences instead of objective exposure measurements, they provide useful descriptive evidence to inform work-place health surveillance and job based preventive strategies.

Within the studied context, the findings support the need for task-oriented interventions such as ergonomic modification of workstations, reduction of repetitive manual tasks through appropriate mechanization, improvement of ventilation and dust control, mitigation of noise and vibration, provision of suitable personal protective equipment, and structured rest breaks. Strengthening routine occupational health monitoring may help address symptom burden among workers in specific job categories.

Overall, this study contributes context-specific evidence on job-related patterns of perceived health complaints in Ayurvedic pharmaceutical manufacturing units and underscores the value of job-focused approaches for improving working conditions in labor-intensive traditional production systems. Further studies involving a larger number of facilities and objective exposure assessment are required to strengthen external validity and extend these findings to other settings.

## 6 Limitation and future prospects

Since this study is a cross-sectional survey, it limits its ability to establish causal relationship between occupational exposure and health outcomes. The association observed here reflects the conditions of the workers at a single point in time and does not capture temporal changes in exposure or health status. The reliance on self-reported health symptoms and perceived workplace exposures to factors such as noise, dust, or temperature may cause reporting bias or recall bias. Underreporting or normalization of symptoms, especially in mental health outcomes since psychological symptoms are known to be less readily disclosed in an occupational setting.

This study was conducted in a limited number of Ayurvedic pharmaceutical manufacturing unit selected using lottery method. However, workers within were selected using convenience sampling. Consequently, while unit level selection biased was minimized, the findings may not be representative of all Ayurvedic pharmaceutical units or workers.

Pre-existing medical conditions were assessed through self-report and were not clinically verified. Residual confounding from unmeasured non-occupational factors, including lifestyle behaviors and underlying health conditions, cannot be excluded. Therefore, the findings should be interpreted as demonstrating associations rather than causal relationships.

Future research should be a longitudinal study design to better understand the long-term health effects of sustained occupational exposure in an Ayurvedic pharmaceutical manufacturing unit.

Objective ergonomic assessment and environmental monitoring were not performed. Therefore, the the findings reflect self-reported symptoms and perceived exposure rather than actual exposure. Future studies should include detailed investigation using objective ergonomic assessment and environmental monitoring of dust, noise, temperature, and chemical constituents to identify actual exposures and health-risks at workplace to strengthen exposure characterization.

Further studies to investigate the association between specific work practices in an Ayurvedic manufacturing unit and exposures with health outcomes like fatigue, respiratory problems, skin diseases and bladder/bowel dysfunction must be conducted. In addition, focused investigations are required to assess the health effects of exposure to metals and minerals among workers involved in rasaushadhi manufacturing, as well as potential risk associated with prolonged exposure to Ayurvedic medicinal herbs and chemicals. Such studies will help strengthen occupational health guidelines and support in development of sector specific regulatory standards.

## Data Availability

The raw data supporting the conclusions of this article will be made available by the authors, without undue reservation.
