# Supplementary material for: Job specific health status of workers in ayurvedic pharmaceutical manufacturing units across Kerala: a cross-sectional study
Source: Front Public Health. 2026 Mar 11;14:1781213. doi: 10.3389/fpubh.2026.1781213 (PMC13013435; doi:10.3389/fpubh.2026.1781213)
Supplement: Supplementary file 1 [file Data_Sheet_1.pdf]

Questionnaire

Title:

"A Cross-sectional Survey to Assess the Health Status of Workers in Medium and Small Scale Ayurvedic Pharmaceutical Industries across Kerala."

1. **Section 1: Demographic information**
   1) Name:
   2) Age:
   3) Sex: Male ☐ Female ☐
   4) Religion: Hindu ☐ Christian ☐
      Muslim ☐ Others ☐
   5) Marital status: Married ☐ Unmarried ☐ Widow/widower ☐
      Divorced ☐
   6) Position / job title:
   7) Years of experience in Ayurvedic pharmaceutical industry:
   8) Height: Weight: BMI:
   9) Preplacement and periodic data:

Section 2: Physical Status of Health

The questions in this section ask about your physical health status after joining the industry.

10) Do you feel tiredness?

a) Never ☐
b) Rarely ☐
c) Sometimes ☐
d) Often ☐
e) Always ☐

11) Do you suffer from nasal complaints? (blocked nose, running nose or sneezing fits)

a) Never ☐
b) Rarely ☐
c) Sometimes ☐
d) Often ☐

e) Always ☐

12) Do you suffer from respiratory problems? (coughing, wheezing, or breathing difficulties)

a) Never ☐
b) Rarely ☐
c) Sometimes ☐
d) Often ☐
e) Always ☐

13) Do you suffer from allergies or skin diseases? (eczema or skin rashes)

a) Never ☐
b) Rarely ☐
c) Sometimes ☐
d) Often ☐
e) Always ☐

14) Do you ever suffer from irritation of your eyes such as pricking, itching, burning, dryness, watering, soreness or stinging of the eyes?

a) Never ☐
b) Rarely ☐
c) Sometimes ☐
d) Often ☐
e) Always ☐

15) Do you ever suppress the urges like micturition, defecation during working time?

a) Never ☐
b) Rarely ☐
c) Sometimes ☐
d) Often ☐
e) Always ☐

16) How often do you experience pain while engaging in work related activities?

a) Never ☐
b) Rarely ☐
c) Sometimes ☐
d) Often ☐
e) Always ☐

17) Do you require standing for a prolonged duration during working hours?

a) Never ☐
b) Rarely ☐
c) Sometimes ☐
d) Often ☐
e) Always ☐

18) Do you have a need to perform repetitive movements with your hands or wrists during working hours?

a) Never ☐
b) Rarely ☐
c) Sometimes ☐
d) Often ☐
e) Always ☐

19) Do you lift, carry or push items manually, heavier than 20kg more than 10 times during the working hours?

a) Never ☐
b) Rarely ☐
c) Sometimes ☐
d) Often ☐
e) Always ☐

20) Do you require to sit continuously for more than 5 hours without a break during working hours?

a) Never ☐
b) Rarely ☐
c) Sometimes ☐
d) Often ☐
e) Always ☐

21) Do you require bending down as part of your work?

a) Never ☐
b) Rarely ☐
c) Sometimes ☐
d) Often ☐
e) Always ☐

22) How frequently does the nature of your job or the environment in which you work directly lead to instances where you have to take sick leave or be absent from work due to illness or health issues?

a) Never ☐
b) Rarely ☐
c) Sometimes ☐
d) Often ☐
e) Always ☐

<u>Section 3: Mental Health Status</u>

The questions in this section ask about your mental health and health-related behaviour.

23) Do you feel excessive stress during work time?

a) Never ☐
b) Rarely ☐
c) Sometimes ☐
d) Often ☐
e) Always ☐

24) Do you often find it difficult to maintain focus or concentration during your work hours?

a) Never ☐
b) Rarely ☐
c) Sometimes ☐
d) Often ☐
e) Always ☐

25) Do you experience moodiness or temper or angry outburst?

a) Never ☐
b) Rarely ☐
c) Sometimes ☐
d) Often ☐
e) Always ☐

26) Do you experience disturbed sleep?

a) Never ☐
b) Rarely ☐
c) Sometimes ☐
d) Often ☐
e) Always ☐

27) Do you find difficulty in falling asleep?

a) Never ☐
b) Rarely ☐
c) Sometimes ☐
d) Often ☐
e) Always ☐

Section 4: Working Environment

The questions in this section ask about physical characteristics of your work environment

28) During work, are you exposed to too much noise?

a) Never ☐
b) Rarely ☐
c) Sometimes ☐
d) Often ☐
e) Always ☐

29) Do you regularly have to raise your voice at work in order to be heard?

a) Never ☐
b) Rarely ☐
c) Sometimes ☐
d) Often ☐
e) Always ☐

30) Do you suffer from the effects of mechanical vibrations or shocks during working hours?

a) Never ☐
b) Rarely ☐
c) Sometimes ☐
d) Often ☐
e) Always ☐

31) How often are you exposed to temperature?

a) Never ☐
b) Rarely ☐
c) Sometimes ☐
d) Often ☐
e) Always ☐

32) How often are you exposed to sun light?

a) Never ☐
b) Rarely ☐
c) Sometimes ☐
d) Often ☐
e) Always ☐

33) How often are you exposed to dust?

a) Never ☐
b) Rarely ☐
c) Sometimes ☐
d) Often ☐
f) Always ☐

34) How often are you exposed to smoke?

a) Never ☐
b) Rarely ☐
c) Sometimes ☐
d) Often ☐
g) Always ☐
