# Supplementary material for: Job specific health status of workers in ayurvedic pharmaceutical manufacturing units across Kerala: a cross-sectional study
Source: Front Public Health. 2026 Mar 11;14:1781213. doi: 10.3389/fpubh.2026.1781213 (PMC13013435; doi:10.3389/fpubh.2026.1781213)
Supplement: Supplementary file 2 [file Data_Sheet_2.pdf]

Exploratory factor analysis (EFA)

Variable	Items	KMO and Bartlett's tests of Sphericity	Factor Loadings	Cronbach's Alpha
Physical Status of Health	Q1	KMO = 0.775<br>Chi-square = 200.961<br>Sig. = 0.000	0.841	0.874
	Q3		0.678	
	Q5		0.933	
	Q6		0.68	
	Q7		0.834	
	Q8		0.982	
	Q10		0.856	
	Q11		0.886	
	Q12		0.642	
Mental Status of Health	Q14	KMO = 0.775<br>Chi-square = 52.362<br>Sig.=0.000	0.887	0.791
	Q15		0.717	
	Q16		0.901	
	Q17		0.663	
Working environment	Q19	KMO=0.555<br>Chi-square= 59.999<br>Sig.= 0.000	0.621	0.841
	Q20		0.621	
	Q22		0.958	
	Q23		0.841	
	Q24		0.941	
	Q25		0.987	

Confirmatory factor analysis (CFA)

Variable	Items	Average Variance Extracted	Composite Reliability
Physical Status of Health	Q1	0.677	0.949
	Q3		
	Q5		
	Q6		
	Q7		
	Q8		
	Q10		
	Q11		
	Q12		
Mental Status of Health	Q14	0.638	0.874
	Q15		
	Q16		
	Q17		
Working environment	Q19	0.688	0.926
	Q20		
	Q22		
	Q23		
	Q24		
	Q25		
